# Improving the efficiency of feed utilization in poultry by selection. 1. Genetic parameters of anatomy of the gastro-intestinal tract and digestive efficiency

**DOI:** 10.1186/1471-2156-12-59

**Published:** 2011-07-06

**Authors:** Hugues de Verdal, Agnès Narcy, Denis Bastianelli, Hervé Chapuis, Nathalie Même, Séverine Urvoix, Elisabeth Le Bihan-Duval, Sandrine Mignon-Grasteau

**Affiliations:** 1 INRA, UR83 Recherches Avicoles, F-37380, Nouzilly, France; 2 CIRAD, UMR SELMET, 34398 Montpellier cedex 5, France; 3 SYSAAF, UR83 Recherches Avicoles, F-37380, Nouzilly, France

## Abstract

**Background:**

Feed costs represent about 70% of the costs of raising broilers. The main way to decrease these costs is to improve feed efficiency by modification of diet formulation, but one other possibility would be to use genetic selection. Understanding the genetic architecture of the gastro-intestinal tract (GIT) and the impact of the selection criterion on the GIT would be of particular interest. We therefore studied the genetic parameters of AMEn (Apparent metabolisable energy corrected for zero nitrogen balance), feed efficiency, and GIT traits in chickens.

Genetic parameters were estimated for 630 broiler chickens of the eighth generation of a divergent selection experiment on AMEn. Birds were reared until 23 d of age and fed a wheat-based diet. The traits measured were body weight (BW), feed conversion ratio (FCR), AMEn, weights of crop, liver, gizzard and proventriculus, and weight, length and density of the duodenum, jejunum and ileum.

**Results:**

The heritability estimates of BW, FCR and AMEn were moderate. The heritability estimates were higher for the GIT characteristics except for the weights of the proventriculus and liver. Gizzard weight was negatively correlated with density (weight to length ratio) of duodenum, jejunum and ileum. Proventriculus and gizzard weights were more strongly correlated with AMEn than with FCR, which was not the case for intestine weight and density.

**Conclusions:**

GIT traits were largely dependent on genetics and that selecting on AMEn or FCR would modify them. Phenotypic observations carried out in the divergent lines selected on AMEn were consistent with estimated genetic correlations between AMEn and GIT traits.

## Background

Improving feed efficiency is a major factor in reducing the costs of poultry production and the environmental impact of chicken production. Many genetic studies have shown that feed efficiency could be improved by selecting on growth, FCR (feed conversion ratio) or feed intake, which are heritable [1]. Mignon-Grasteau *et al*. [2] recently showed that the ability of the animal to digest its feed could also be used as a selection criterion. Following this study, two lines (D+ and D-) were divergently selected on digestive efficiency assessed by the AMEn (Apparent Metabolisable Energy corrected for zero nitrogen retention) of a wheat-based diet, measured at 3 weeks of age. After 7 generations of selection, D+ and D- lines differed by about 30 to 40% on the selection criterion, but presented similar body weights [3]. Mignon-Grasteau *et al*. [2] and Rougière *et al*. [4] also showed that levels of starch, protein, and lipid digestibility were greater for D+ birds compared to D- birds. These lines also differed in feed consumption which was much higher in D- [5] and in the morphology of the digestive tract [4,6] since D+ birds presented a heavier proventriculus and gizzard and a lighter, shorter and less dense small intestine than D- birds at 23 d of age. These strongly correlated changes of gastro-intestinal tract organs suggested that both these traits were heritable and genetically correlated with AMEn.

The aim of the present study was therefore to understand the impact of the selection criterion (AMEn, FCR or residual feed intake (RFI)), on the characteristics of the GIT. Estimation of the genetic parameters should make it possible to establish how selection impacts on the morphology of the gut organs and to offer the opportunity to anticipate any undesirable effects of AMEn selection.

## Results

### Between-line differences

The elementary statistics for performance traits and gastro-intestinal morphology for both lines are reported in Table 1. The sex effect was significant for BW23, FCR, RFI, crop weight, duodenum length and density. Males were 7.6% heavier and presented a 9.0% higher FCR than females. The residual consumption was 15% higher for females than for males. A line effect was significant for all traits, except for crop weight. Indeed, the D+ birds had a 33.5% higher AMEn, a 14.5% higher BW23, a 13.7% higher WG and a 21.5% lower FI than D- birds. This suggests that the 36.8% difference in FCR between D+ and D- birds could be attributed to two-thirds to FI and one-third to WG differences. The difference in RFI between D+ and D- represented 21% of the average consumption. Of the GIT traits, relative gizzard and proventriculus weights were greater in D+ birds, and the jejunum and ileum were smaller, shorter, and less dense compared to D- birds. The proventriculus and gizzard weights were 21.9% and 34.0% higher in D+ compared to D- birds, respectively. In contrast, the liver and the small intestine trait values (relative weight, relative length, and density) were higher in D- than in D+ birds. Differences were minor for relative liver weight and relative length of intestinal segments (between 2.7 and 6.0%), moderate for relative duodenum weight and density (between 10.3 and 13.0%), but much greater for jejunum and ileum relative weights and densities (between 23.1 and 28.5%). Including FI as covariate in the model of analysis of gastro-intestinal tract characteristics did not change the results as compared to the model without FI covariate.

**Table 1:** Basic statistics (LS Means ± Standard Deviation (Coefficients of Variation)) for all traits analysed (N ranging from 570 to 598 according to the trait).

Trait	D+	D-	D+/D-ratio (%)	Significance of line effect
BW23^1^	490 ± 64.5 (13.2)	428 ± 64.9 (15.2)	14.5	< 0.001
WG	166 ± 30.6 (18.4)	146 ± 28.9 (19.8)	13.7	< 0.001
FI	285 ± 41.8 (14.7)	363 ± 66.6 (18.3)	-21.5	< 0.001
AMEn	3,278 ± 148 (4.52)	2,455 ± 533 (21.7)	33.5	< 0.001
FCR	1.72 ± 0.39 (22.7)	2.72 ± 0.54 (19.9)	-36.8	< 0.001
RFI	-33.2 ± 3.15 (9.49)	34.9 ± 3.2 (9.17)	-^5^	< 0.001

*Weight*^2^				
Crop	4.78 ± 0.75 (15.7)	4.74 ± 0.77 (16.2)	0.84	0.620
Proventriculus	8.19 ± 2.08 (25.4)	6.72 ± 1.39 (20.7)	21.9	< 0.001
Gizzard	26.0 ± 4.2 (16.2)	19.4 ± 4.23 (21.8)	34.0	< 0.001
Liver	34.0 ± 3.81 (11.2)	34.9 ± 3.67 (10.5)	-2.58	0.003
Duodenum	12.7 ± 2.38 (18.7)	14.6 ± 2.32 (15.9)	-13.0	< 0.001
Jejunum	17.2 ± 2.53 (14.7)	23.5 ± 3.46 (14.7)	-26.8	< 0.001
Ileum	12.8 ± 1.98 (15.5)	17.9 ± 2.74 (15.3)	-28.5	< 0.001

*Length*^3^				
Duodenum	49.7 ± 7.45 (15.0)	51.1 ± 7.45 (14.6)	-2.74	0.030
Jejunum	87.7 ± 15.6 (17.8)	93.3 ± 14.9 (16.0)	-6.00	< 0.001
Ileum	84.2 ± 16.1 (19.1)	87.4 ± 14.7 (16.8)	-3.66	0.019

*Density*^4^				
Duodenum	0.26 ± 0.04 (15.4)	0.29 ± 0.04 (13.8)	-10.3	< 0.001
Jejunum	0.20 ± 0.04 (20.0)	0.26 ± 0.04 (15.4)	-23.1	< 0.001
Ileum	0.16 ± 0.03 (18.8)	0.21 ± 0.04 (19.0)	-23.8	< 0.001

^1 ^BW23, body weight at 23 d (g); WG, body weight gain between 17 and 23 d (g); FI, feed intake between 17 and 23 d (g); AMEn, apparent metabolisable energy corrected for zero nitrogen balance (kcal.kg^-1 ^DM); FCR, feed conversion ratio between 17 and 23 d (kg.kg^-1^); RFI, residual feed intake (g)

^2 ^Weight, organ weight in relation to BW23 (g.kg^-1^);

^3 ^Length, organ length in relation to BW23 (cm.kg^-1^);

^4 ^Density, weight to length ratio (g.cm^-1^) of the different segments of the GIT

^5 ^In this case, the difference between the lines is not relevant

### Genetic parameters of performance and feed efficiency

The genetic parameters of performance traits are shown in Table 2. Heritability estimates were moderate to high, ranging between 0.21 ± 0.02 for FCR and 0.47 ± 0.05 for FI. Maternal permanent environmental effects were estimated at 0.15 ± 0.01, 0.08 ± 0.01, and 0.06 ± 0.01 for BW23, AMEn and FCR, respectively.

**Table 2:** Estimated genetic parameters (± standard errors) for body weight at 23 d (BW23), body weight gain (WG), feed intake (FI), apparent metabolisable energy (AMEn), feed conversion ratio (FCR), and residual feed intake (RFI).

Trait^1^	BW23	WG	FI	AMEn	FCR	RFI
BW23	**0.28 ± 0.04** *0.15 *± *0.01*	0.99 ± 0.01	0.66 ± 0.06	0.24 ± 0.06	-0.24 ± 0.06	0.70 ± 0.05
WG		**0.30 ± 0.06**	0.60 ± ne^2^	0.24 ± 0.07	-0.35 ± ne	0.54 ± ne
FI			**0.47 ± 0.05**	-0.66 ± 0.05	0.64 ± ne	0.99 ± ne
AMEn				**0.30 ± 0.02** *0.08 *± *0.01*	-0.98 ± 0.02	-0.60 ± 0.06
FCR					**0.21 ± 0.02** *0.06 *± *0.01*	0.57 ± ne
RFI						**0.46 ± 0.06**

^1 ^Estimates of heritability (h^2^) on the diagonal in bold; maternal permanent environmental effects (c^2^) on diagonal in italics; genetic correlations (r_g_) above diagonal. ^2 ^ne: not estimated.

The genetic correlation between FCR and AMEn was not significantly different from unity. This was also the case for the genetic correlations between FI and RFI, and between BW23 and WG. AMEn and RFI were significantly negatively correlated and, in contrast, FCR and RFI were positively correlated. The BW23 was poorly and negatively correlated with FCR and AMEn, while it was highly and positively correlated with RFI (0.70 ± 0.05).

### Genetic parameters of GIT traits

The genetic parameters of the characteristics of the GIT morphology are presented in Table 3. Heritability estimates were very low and not significantly above zero for proventriculus and liver weights. Crop weight was found to be moderately heritable (0.21 ± 0.06). For all other GIT traits, heritability estimates were high (from 0.28 ± 0.06 for ileum length to 0.53 ± 0.11 for gizzard weight). The maternal permanent environmental effect was found to influence the relative proventriculus and gizzard weights (c^2 ^= 0.19 ± 0.03 and 0.12 ± 0.05, respectively) but not the characteristics of the other components of the GIT.

**Table 3:** Estimates (± standard errors) of heritability (on diagonal) and genetic correlations (above diagonal) for GIT traits

Trait^1^	CW	PRW	GZW	LW	DW	JW	IW	DL	JL	IL	DD	JD	ID
CW	0.21 ± 0.06	0.53 ± 0.14	0.09 ± 0.06	0.75 ± 0.13	0.18 ± 0.08	-0.14 ± 0.13	0.04 ± 0.10	0.23 ± 0.19	-0.13 ± ne^2^	-0.10 ± 0.23	-0.05 ± 0.20	0.11 ± 0.19	0.22 ± 0.18
PRW		0.09 ± 0.06	0.81 ± 0.15	-0.55 ± ne	0.54 ± 0.20	-0.28 ± 0.40	0.17 ± 0.19	0.51 ± 0.43	0.52 ± 0.40	0.65 ± 0.29	-0.18 ± 0.26	-0.56 ± 0.20	-0.32 ± 0.27
GZW			0.53 ± 0.11	-0.51 ± 0.30	0.13 ± 0.16	-0.25 ± 0.16	-0.03 ± 0.14	0.16 ± ne	0.28 ± 0.15	0.22 ± 0.17	-0.37 ± 0.12	-0.56 ± 0.13	-0.33 ± 0.11
LW				0.05 ± 0.02	0.38 ± 0.35	0.66 ± 0.20	0.65 ± 0.25	0.70 ± 0.09	0.75 ± 0.08	0.24 ± 0.27	-0.47 ± 0.21	-0.37 ± 0.20	-0.03 ± 0.21
DW					0.33 ± 0.06	0.62 ± 0.08	0.70 ± 0.06	0.67 ± ne	0.39 ± 0.14	0.52 ± 0.11	0.38 ± ne	0.17 ± 0.13	0.20 ± 0.13
JW						0.44 ± 0.06	0.88 ± 0.03	0.40 ± 0.12	0.21 ± 0.15	0.15 ± ne	0.21 ± 0.13	0.60 ± 0.09	0.60 ± ne
IW							0.37 ± 0.05	0.33 ± ne	0.16 ± 0.13	0.21 ± ne	0.41 ± 0.14	0.54 ± 0.11	0.67 ± 0.08
DL								0.46 ± 0.06	0.89 ± ne	0.93 ± ne	-0.48 ± ne	-0.34 ± 0.13	-0.40 ± 0.12
JL									0.32 ± 0.06	0.90 ± 0.04	-0.71 ± 0.09	-0.64 ± 0.09	-0.61 ± 0.09
IL										0.28 ± 0.06	-0.52 ± 0.12	-0.56 ± ne	-0.57 ± 0.10
DD											0.39 ± 0.06	0.78 ± 0.05	0.79 ± 0.06
JD												0.50 ± 0.07	0.93 ± 0.02
ID													0.44 ± 0.06

^1^CW, PRW, GZW, LW, DW, JW, IW, weights of crop, proventriculus, gizzard, liver, duodenum, jejunum, and ileum in relation to BW; DL, JL, IL, lengths of duodenum, jejunum and ileum in relation to BW; DD, JD, ID, density of duodenum, jejunum and ileum. ^2 ^ne: not estimated.

Correlations between components of the upper part of the GIT were heterogeneous. Crop weight was correlated with proventriculus but not gizzard weight (0.53 and 0.09, respectively). In contrast, a strong positive genetic correlation was found between proventriculus and gizzard weights (0.81).

Whatever the measurement (weight, length or density), the three segments of the small intestine exhibited strong genetic correlations, slightly lower between the duodenum and the other two segments (ranging from 0.62 to 0.93) than between the jejunum and ileum (from 0.88 to 0.93). Intestinal density was strongly positively correlated with relative weights of segments and negatively with their relative lengths, with slightly lower values for the duodenum than for the jejunum and ileum. Conversely, the genetic correlations between relative intestinal weight and length were greater for the duodenum (r_g _= 0.67) than for the jejunum and ileum (r_g _= 0.21). Finally, it was of note that correlations between liver weight and intestinal traits were also heterogeneous between segments, being strong and positive with JL, JW, IW and DL, but low to moderate with densities, DW and IL.

Liver weight was found to be strongly positively correlated with crop weight, but negatively with GW. Crop and gizzard weights were uncorrelated with weight, length and density of intestinal segments, except for a negative correlation between GZW and JD (-0.56). In contrast, relative proventriculus weight was positively correlated with intestine lengths, DW and JD, absolute values ranging between 0.51 and 0.65.

### Genetic correlations between digestibility and anatomy of the GIT

The genetic correlations between the digestibility, performance and feed efficiency and the GIT morphologic characteristics are shown in Table 4. Relative crop weight was not correlated with the performance traits. BW23 was moderately to strongly correlated with all other traits, especially with relative intestine lengths, LW and DD (-0.94 to -0.96, -0.90 and 0.76, respectively). Consistent with the genetic correlation between BW23 and WG (0.99), the genetic correlations between WG and the GIT morphology traits were similar to those between BW23 and the GIT characteristics, except for the intestinal density, where the genetic correlations were relatively lower with WG than with BW23. Interestingly, AMEn and RFI were more highly correlated with relative proventriculus and gizzard weights (from 0.43 to -0.67) than FCR (-0.19 and -0.25). In contrast, AMEn and FCR showed similar patterns of correlation with intestinal lengths and weights relative to BW23 and densities, whereas RFI and FI showed a different pattern. Indeed, AMEn and FCR were not correlated with relative intestinal lengths but moderately to highly with relative intestinal weights (-0.36 to -0.77 and 0.36 to 0.66, for AMEn and FCR, respectively), whereas RFI and FI were uncorrelated with relative weights but strongly correlated with relative lengths (-0.46 to - 0.63). Finally, AMEn, FCR, FI and RFI were all moderately correlated with densities, with absolute genetic correlations increasing from the duodenum to the ileum, at least for AMEn and FCR.

**Table 4:** Genetic correlations (± standard errors) between body weight at 23 d (BW23), body weight gain (WG), feed intake (FI), apparent metabolisable energy (AMEn), feed conversion ratio (FCR) and residual feed intake (RFI) and characteristics of GIT morphology.

Trait^1^	BW23	WG	FI	AMEn	FCR	RFI
CW	-0.18 ± 0.21	-0.16 ± 0.21	0.19 ± 0.18	0.05 ± 0.09	0.18 ± 0.20	0.24 ± 0.18
PRW	-0.53 ± 0.28	-0.75 ± 0.11	-0.11 ± ne	0.59 ± 0.22	-0.25 ± ne^2^	-0.67 ± 0.48
GZW	-0.45 ± 0.15	-0.51 ± ne	-0.36 ± 0.15	0.43 ± 0.12	-0.19 ± ne	-0.47 ± ne
LW	-0.90 ± 0.10	-0.99 ± ne	-0.64 ± ne	-0.49 ± 0.18	0.53 ± 0.28	-0.32 ± 0.16
DW	-0.50 ± 0.15	-0.50 ± 0.12	0.08 ± 0.13	-0.36 ± 0.08	0.36 ± 0.09	0.02 ± 0.14
JW	-0.45 ± 0.15	-0.54 ± 0.13	0.05 ± 0.12	-0.67 ± 0.07	0.55 ± 0.09	0.02 ± 0.13
IW	-0.42 ± 0.13	-0.63 ± 0.10	0.03 ± 0.10	-0.77 ± 0.06	0.66 ± 0.08	-0.03 ± ne

DL	-0.94 ± 0.02	-0.83 ± 0.08	-0.46 ± ne	-0.05 ± 0.07	-0.05 ± ne	-0.47 ± 0.10
JL	-0.96 ± 0.02	-0.90 ± ne	-0.58 ± ne	-0.10 ± 0.07	0.07 ± 0.09	-0.60 ± 0.09
IL	-0.95 ± 0.02	-0.98 ± 0.01	-0.60 ± 0.10	-0.05 ± 0.07	0.01 ± ne	-0.63 ± ne

DD	0.76 ± 0.08	0.37 ± ne	0.58 ± ne	-0.30 ± 0.07	0.25 ± 0.08	0.54 ± 0.10
JD	0.60 ± 0.13	0.28 ± ne	0.49 ± ne	-0.39 ± 0.07	0.32 ± 0.07	0.51 ± 0.10
ID	0.58 ± 0.10	0.25 ± ne	0.52 ± ne	-0.52 ± 0.07	0.47 ± 0.07	0.53 ± 0.10

^1^CW, PRW, GZW, LW, DW, JW, IW, weights of crop, proventriculus, gizzard, liver, duodenum, jejunum and ileum in relation to BW; DL, JL, IL, lengths of duodenum, jejunum and ileum in relation to BW; DD, JD, ID, density of duodenum, jejunum and ileum. ^2 ^ne: not estimated.

## Discussion

### Heritability estimates

Our estimates of the genetic parameters of performance traits were relatively similar to those of Mignon-Grasteau *et al*. [2] on the same lines. However, we found lower levels of heritability for BW23, FCR and AMEn, probably as our model of analysis included an environmental permanent maternal effect, which was not the case in the earlier study. For RFI the present estimate was in accordance with previous results showing similar estimates at this age [2] and between 28 and 35 d [7]. Due to the complexity of measurements, very few studies have presented genetic parameters of GIT characteristics [8,9]. The present heritability estimate for the liver weight relative to BW23 was close to the value of 0.11 obtained by Rance *et al*. [9]. However, these estimates were low compared to certain studies performed in poultry [8,10]. These authors estimated liver weight heritability at 0.27 and 0.25, respectively, but worked on absolute and not relative liver weights. Moreover, they did not consider the maternal environmental effect that we estimated at 0.09 ± 0.02. In addition, Ledur *et al*. [10] and Gaya *et al*. [8] used commercial broilers at 42 d of age, whereas our genotypes exhibited slower growth and were measured at 23 d of age, probably explaining the extent of the maternal effects. The same hypothesis is advanced to explain the low heritability of the relative proventriculus weight (0.09 ± 0.06) compared to the literature [9]. With our data, excluding the permanent environment effects in the analysis led to a substantial overestimation of heritability of the proventriculus (i.e., 0.63).

The heritability of the relative gizzard weight was high (0.53 ± 0.11) even when environmental permanent maternal effects were included in the model. This estimate is consistent with previous estimates of Gaya *et al*. [8] and Rance *et al*. [9] for the absolute gizzard weight (0.39 to 0.52).

In the study presented here, heritability of the relative intestine weight was high compared to the estimates of Rance *et al*. [9] and Gaya *et al*. [8], which ranged between 0.00 and 0.29 for the relative jejunum weight [9] and the small intestine weight [8], respectively. The fact that we worked on relative and not on absolute values could not explain these differences, since Rance *et al*. [9] found similar heritability estimates for relative and absolute values. A more probable explanation of the difference between these two studies and our estimates is the difference in age at measurement, i.e. 23 d in our case and 42 d in their studies [8,9]. At 23 d, the development of the intestine is exponential, which is no longer the case at 42 d. Furthermore, Rance *et al*. [9] and Gaya *et al*. [8] used a conventional corn-based diet, whereas we used a low digestibility wheat-based diet. Mignon-Grasteau *et al*. [11] estimated that the heritability of traits was much lower with a corn-based diet than with the latter diet, as the wheat diet enhanced differences between animals. The relative lengths and densities of the 3 segments of the small intestine were thus highly heritable (from 0.28 ± 0.06 to 0.50 ± 0.07).

### Genetic correlations

#### *Between the GIT components*

Very few estimates of genetic correlations are available in the literature due to the complexity of measurement. Relative proventriculus and gizzard weights were highly genetically correlated, as in Rance *et al*. (0.59 [9]). This correlation is consistent with the evolution of the gizzard and the proventriculus observed in both lines. Furthermore, it could be expected since from an anatomical and physiological point of view the proventriculus and the gizzard are linked and have complementary roles in the pre-digestion process, conditioning the availability of nutrients in the small intestine.

In our study, the relative gizzard weight was negatively correlated with intestine density. In agreement with this, Nir *et al*. [12] had already shown that chicken developed a larger gizzard and a lighter intestine with a coarse diet than with a fine diet. When whole wheat was added to a wheat-based diet, Wu *et al*. [13] showed that digesta viscosity was increased in the duodenum and the jejunum in parallel with an increase in absorption rates and a reduction in intestinal density [14]. For both lines in our study, jejunum and ileum weights relative to BW23 and densities were 30.0 to 39.8% lower in D+ birds, as previously shown [4,6]. Furthermore, de Verdal *et al*. [6] showed histologically that the absorptive epithelium and the muscle layer of the small intestine were thicker in D- than in D+ birds, thus explaining, at least in part, the greater intestine weight in D- than in D+ birds. These authors hypothesized that the small intestine grew in response to the functional efficiency of the gizzard.

The genetic correlations between the three intestinal segments estimated for the relative weights, relative lengths and densities were quite high (0.62 to 0.93), in accordance with the results of Rance *et al*. [9] who estimated the genetic correlations between the duodenum and the ileum weights to be 0.98. This therefore implies that the 3 intestinal segments show similar modifications, consistent with the phenotypic differences recently observed in D+ and D- lines, indicating that the AMEn selection affected the whole small intestine [6]. Moreover, the phenotypic differences between the two divergent lines were higher for the relative intestine weights than for the relative intestine lengths. This is in accordance with the observation that, in contrast to the relative intestine lengths, the relative intestine weights were highly correlated with AMEn. The genetic correlations were therefore greater between the jejunum and the ileum than between the duodenum and the other two segments, in accordance with previous phenotypic results. Indeed, histological differences between D+ and D- lines were greater in the jejunum and ileum than in the duodenum [6]. Furthermore, this could be explained by functional differences between the intestinal segments. Absorption processes predominate in the jejunum and ileum [15] and the digestive environment is different between the segments, including pH and enzymes [16,17]. The levels of α-amylase and trypsin activity were higher in the jejunum and ileum [18], where absorption takes place, than in the duodenum. Moreover, according to some authors, post-hatch development is different between the segments: small changes occur in the duodenum length after 7 d, whereas the jejunum and ileum lengths continue to increase after 2 weeks of age [19].

#### *Between AMEn and GIT compartments*

Selection on AMEn involved differential development of the digestive tract between the divergent lines. The relative proventriculus and gizzard were 21.9% and 34.0% heavier in D+ than in D- birds. The positive correlations between relative proventriculus and gizzard weights and AMEn, the selection criterion, are consistent with previous phenotypic correlations on these lines [20] and fast and slow growing lines [21]. Moreover, it had already been shown that a larger gizzard was associated with better starch and protein digestibility [22,23], genetically highly correlated with the AMEn (0.83 ± 0.03 and 0.86 ± 0.03, respectively) according to Mignon-Grasteau *et al*. [2]. In agreement with this, D+ birds have a 10 and 13% better starch and protein digestibility, respectively, than D- birds [11]. Ravindran *et al*. [24] and Gonzalez Alvarado *et al*. [16] showed that a moderate development of the gizzard was related to low levels of digestibility. These findings confirmed the central role of the upper part of the digestive tract in the digestion process. Indeed, greater proventriculus and gizzard development enhances grinding of the digesta, hydrogen chloride and pepsinogen secretion by the proventriculus and reflux from the duodenum [25]. Additionally, Rougière and Carré [5] showed that the mean retention time in the proventriculus and gizzard of D- was lower than in D+ birds, which may improve nutrient accessibility and absorption. Furthermore, AMEn, and relative proventriculus and gizzard weights were negatively genetically correlated with the relative liver weight. According to Mignon-Grasteau *et al *[2], D+ birds present higher levels of lipid digestibility (+23.6%) compared to D- birds. This suggests that although D+ birds showed lower relative liver weights, the amounts of bile salts synthesized appear not to limit lipid digestibility.

The negative genetic correlations between the relative weights of the intestine and AMEn were consistent with phenotypic observations [20,26]. Taylor & Jones [14] have already reported improved absorption when intestinal density is decreased. Furthermore, Mitchell & Smith [27] showed that a decrease in the intestinal mucosal mass resulted in more efficient activity of the small intestine. Part of the explanation may be that the reduction in intestinal mucosal mass in D+ birds may result in lower energy expenditure [27]. Positive genetic correlations between the relative intestine weights and FCR were also found by Gaya *et al*. [8], consistent with the phenotypic modifications in D+ and D- lines [6]. Furthermore, the results of the analysis of covariance with FI as covariate confirmed the fact that the quantity of feed passing through the gastro-intestinal tract cannot be seen as the only cause of differences between lines.

The selection experiment was performed at 23 d of age because it represents a key period in the GIT development [28]. In addition, the differences between lines for feed efficiency were still observed at 53 d of age, when birds reached the market weight [3]. Relative weight of gizzard and proventriculus as well as relative weight and density of intestinal segments presented similar differences between lines at 53 d and at 23 d [3]. Finally, AMEn differences are still observed at 53 d between D+ and D- birds (unpublished data). Thus, conclusions based upon data obtained at 23 d of age would still hold for a whole production cycle.

#### Differences between AMEn, RFI and FCR

D+ birds performed better than D- birds, as shown by the improvement in BW (+14.5%), FCR (-36.8%) and RFI. These results confirmed previous studies showing similar variations in AMEn, RFI and FCR between the two lines [2,29].

The change of the genetic values between the both lines was symmetric, which was not the case of the phenotypic values. Indeed, selection on AMEn reduced more strongly digestibility in the D- birds than it increased in D+ birds. The starch digestibility nearly reached the maximum value of 100%, i.e. 96.3% in D+ *vs *88.4 in D- birds [2]. Besides the increase in digestibility mean value, the D+ birds showed much more homogeneous performances, as indicated by the low coefficient of variation of AMEn in D+ compared to D- birds, which is a criterion of quality in poultry production.

Genetic correlations between AMEn, FCR and RFI were high. The genetic correlation between AMEn and FCR was even stronger than the value of 0.7 found by Mignon-Grasteau *et al*. [2] within the same lines. However, several differences were found in the genetic correlations between AMEn, FCR, RFI and BW and the GIT components.

The genetic correlation between AMEn and BW23 was low but significant, which can explain why we found a difference in BW between D+ and D- lines after 8 generations of selection, whereas no difference was found after one generation of selection by de Verdal *et al*. [3].

Moreover, the strong genetic correlation between RFI and BW indicated that, in contrast to AMEn and FCR, selecting on a reduced RFI would lead to a decrease in BW23 and WG. This is in accordance with previous results in pigs selected on RFI [30], even if our correlation was much higher, probably due to the low r^2 ^of our regression model (0.05). Moreover, reducing RFI will strongly affect FI, as shown by the genetic correlation of 0.99 between both traits.

AMEn is genetically correlated with the weights of the components of the upper (proventriculus and gizzard) and lower (duodenum, jejunum and ileum) parts of the GIT. In contrast, RFI is mainly correlated with the upper part of the GIT, and FCR only with the lower part of the GIT. Consequently, given the importance of the upper and the lower GIT parts and their complementarity in digestive phenomena, selection on AMEn could optimise digestion.

## Conclusions

The present study showed that morphological GIT characteristics were moderately to highly heritable, especially the gizzard and small intestine. This study also showed that the gizzard was genetically linked with intestinal density, consistent with the assumption that events in the upper part of the digestive tract can strongly influence the functionality of the lower part. The phenotypic evolution of lines selected on AMEn is consistent with the present estimates of genetic correlations between anatomy and digestive capacity of birds. The increase in relative gizzard and proventriculus weights in the high AMEn birds probably leads to greater nutrient availability in the small intestine.

In contrast to selection on RFI, selection on AMEn increased BW at 23 d of age. Furthermore, in contrast to FCR or RFI, selection on AMEn had an impact on both the upper and the lower parts of the GIT which together have major complementary roles. The asymmetry of the phenotypic response of AMEn between D+ and D- seems to illustrate that D+ are probably close to the biological limit of 100% of digestibility, especially for starch. Furthermore, the D- birds should be a model to study the limiting steps of nutrients digestion in relation to anatomical and physiological characteristics. Finally, in view of the negative impact of poultry manure on the environment, selection on AMEn would be one way to control excretion. Further studies are needed to quantify precisely the impact of selection on AMEn on excretion characteristics.

## Methods

### Birds and Housing

The experiment was conducted according to the guidelines of the French Ministry of Agriculture for Animal Research. It included 630 birds (307 males and 323 females) of the 8^th ^generation of selection of D+ and D- lines [2], reared in 3 hatches each separated by 4 wk. The pedigree included animals from all generations (i.e., 4495 birds: 122 and 132 sires for D+ and D-, respectively, corresponding to 16.5 and 15.5 offspring per sire for D+ and D-, respectively). They were individually weighed at hatching and placed in groups of 4 or 5 chicks in metal cages (36 cm long × 22 cm wide × 40 cm high) for 3 d. After 3 d, chicks were randomly allocated to individual cages, in 3 different rearing rooms. The environmental conditions were controlled in terms of ventilation, lighting program (24L: 0D from 1 d to 7 d and 23L: 1D from 8 d to 23 d, dark periods beginning at midnight) and temperature (from 33°C at 1 d to 22°C at 23 d). Mortality was recorded daily. The birds had free access to water and food. They were fed a wheat-based diet similar to that used during the selection experiment (Table 5[2]).

**Table 5:** Composition of diet distributed during the rearing period.

Ingredients	Amount (g.kg^-1^)
Corn	60.4
Wheat (Rialto)	525.0
Soybean meal 48	284.0
Corn gluten 60	31.0
Soybean oil	60.0
DL methionine	1.2
L-Lysine 78	2.2
Calcium carbonate	13.4
Dicalcium phosphate	15.8
Sodium chloride	3.0
Mineral and vitamin mix^1^	3.5
Robenidine^2^	0.5

Characteristics^3 ^(calculated)	

AMEn (kcal. kg^-1^)	2 943
Crude proteins (%)	20.5
Lysine (%)	1.16
Methionine + Cystine (%)	0.76
Calcium (%)	1.11
Total phosphorus (%)	0.66
Non-phytate phosphorus (%)	0.42

^1^Supplied per kilogram of diet: 0.5 mg Co; 16 mg Cu; 47 mg Fe; 1.6 mg I; 65 mg Mn; 0.2 mg Se; 72 mg Zn; 12,000 IU retinyl acetate; 3,440 IU cholecalciferol; 80 mg dl-α tocopheryl acetate; 4 mg thiamine; 6.4 mg riboflavin; 20 mg calcium pantothenate; 0.02 mg vitamin B12; 4 mg menadione; 5.6 mg pyridoxine hydrochloride; 0.4 mg folic acid; 0.24 mg biotin; 80 mg niacin; 440 mg choline; 40 mg antioxidant; ^2^Robenz, Alpharma Animal Health, Bridgewater, NJ; ^3^Calculated [36]

### Growth Traits and Morphology of Digestive Tract

All birds were individually weighed at 17 d and 23 d (BW23). The weight gain (WG) between 17 and 23 d of age was calculated. Total individual feed intake (FI) was also recorded from 17 to 23 d and feed conversion ratio during this period (FCR) was calculated. The apparent metabolisable energy corrected for zero nitrogen retention (AMEn) was individually measured between 17 and 23 d using a method based on collection of total excreta, as described by Bourdillon *et al*. [31]. AMEn was measured for all birds using Near Infrared spectrophotometry (NIRS, Foss spectrometer NIRSystems 6500, Inc., Silver Spring, MD) to determine gross energy content of excreta, according to the method of Bastianelli *et al*. [32]. Residual feed intake (RFI) was calculated as the difference between the observed feed intake and the feed intake predicted by regression on BW and BW gain (BWG) between 17 and 23 d, according to the method of Tixier-Boichard *et al*. [33].

At 23 d of age after overnight fasting (8 h) all remaining chicks were sacrificed by CO_2 _inhalation. The crop, liver, proventriculus and gizzard were excised and weighed (CW, LW, PRW, and GZW, respectively). The duodenum (from pylorus to pancreatic loop), jejunum (from the pancreatic loop to Meckel's diverticulum), and ileum (from Meckel's diverticulum to the ileo-caecal junction) were sampled and their lengths measured (DL, JL, and IL, respectively). Segments were then emptied and weighed (DW, JW, and IW, respectively). The weight to length ratio of each segment (DD, JD, and ID, respectively) was also calculated as an indicator of intestine density [14]. Organ weights and lengths were always expressed per kg of BW, corresponding to relative weight and relative length, in order to avoid any confusion between the weights and lengths GIT traits differences and the BW23 differences between D+ and D- birds.

### Statistical Analyses

All data were analyzed according to the General Linear Models (GLM) procedure of SAS [34]. The following model was used:(1)

where y_ijklm _is the performance of animal m, μ the general mean, L_i _the fixed effect of line i (i = D+ or D-), C_j _the effect of rearing room j (j = 1 to 3), H_k _the fixed effect of hatch k (k = 1 to 3), S_l _the fixed effect of sex l, LC_ij _the effect of the interaction between line i and rearing room j, LH_ik _the effect of the interaction between line i and hatch k, and e_ijklm _the residual pertaining to animal m. Least square means and standard deviations were estimated for D+ and D- lines, for each trait. Differences were considered significant when the *P*-value was lower than 0.05. The relative differences between lines were calculated as the ratio of the D+ value to the D- value. In order to check whether the differences in the gastro-intestinal tract morphology between the lines were not due to the differences in feed intake, a covariance analysis was performed with the GLM procedure of SAS [34] including FI in covariate in the analysis model [1].

### Estimation of Genetic parameters

Genetic parameters were estimated by the REML (REstricted Maximum Likelihood) method with VCE4 software [35]. For all estimates, the model used was equation [1] with the addition of the additive genetic effect of animal (N = 4495). Preliminary analyses indicated the presence of a significant maternal effect for BW23, FCR, AMEn, GZW, and PRW. A permanent environmental maternal effect was therefore included in the model for these traits. For BW23, FCR and AMEn, data of the eight generations were included in the analysis in order to take into account the effects of selection in our lines. As several traits were strongly correlated, it was not possible to run a single analysis including all traits. This is the reason why distinct multi-trait analyses were performed, always including traits used in the selection experiment, i.e. AMEn and BW23. A total of 128 analyses were performed. When a parameter (heritability or genetic correlation between two traits) was estimated in several analyses, the parameter estimates and the standard errors of parameters presented below are the means of those obtained in various analyses. Standard errors were not available for several analyses, as several traits presented very high correlations and/or low heritability estimates, preventing the likelihood maximisation algorithm from reaching a single optimum.

## Competing interests

The authors declare that they have no competing interests.

## Authors' contributions

HdV, AN, ELB and SMG contributed to experimental design, data analysis, interpretation of data and manuscript preparation. HC contributed to the data analysis. DB contributed to the use of NIRS in digestibility measurements. NM and SU assisted in the acquisition of data. All authors read and approved the final manuscript.
